# The Importance of Nutrigenetics and Microbiota in Personalized Medicine: From Phenotype to Genotype

**DOI:** 10.7759/cureus.61256

**Published:** 2024-05-28

**Authors:** Gulsen Meral, Elif S Aslan, Verda Tunaligil, Neval Burkay, Esma Gökcen Alper Acar, Muhammed Yunus Alp

**Affiliations:** 1 Molecular Biology and Genetics, Biruni University, Istanbul, TUR; 2 Molecular Biology and Genetics, Epigenetic Coaching, Norwich, GBR; 3 Epidemiology and Public Health, Ministry of Health, Istanbul Health Directorate, The Medical Device and Simulation Center (SIMMERK5), Istanbul, TUR; 4 Nutrition, Epigenetic Coaching, Norwich, GBR; 5 Medical Biology, Merzifon Karamustafa Paşa Devlet Hastanesi, Amasya, TUR; 6 Genetics, Epigenetic Coaching, Norwich, GBR

**Keywords:** personalised approach, methylation, nutrigenomics, nutrigenetic, public health, microbiota

## Abstract

Background

After the completion of the Human Genome Project in 2003, the impact of genetic variations among people on human health was better understood. Precision medicine, also called 4P (Predictive, Preventive, Personalized, Participatory) medicine, is used to determine personal health risks, prevent, diagnose, and treat chronic diseases, and aims to identify the phenotypic, genotypic, and environmental factors that affect individual health risks instead of applying the same approach to everyone.

Methods

The study was conducted with 24 patients aged between 7 and 57. The patient group was selected from individuals who had undergone genetic and microbiota testing at Epigenetic Coaching Company. The patients' age, gender, and health status were documented. Genomic analysis of buccal samples was subsequently conducted using a custom Infinium HTS iSelect microarray on an Illumina iScan instrument, and microbiota metagenome analysis was performed using an Illumina NextSeq 500 platform. This study was performed in line with the principles of the Declaration of Helsinki. Approval was granted by the Ethics Committee of Biruni University Molecular Biology and Genetics Ethics Committee, with the decision number 2023/78-03.

Results

The genotypes of 19 cases carrying genetic variants involved in the metabolism of Vitamin D, Folate, B12, and Choline were analyzed. Eight of the cases were included in our study as autism patients, eight as allergy patients, and three as autoimmune thyroiditis patients. The Vitamin D receptor (VDR) genetic variants and microbiota diversity (using the Firmicutes/Bacteroides ratio, an indicator of dysbiosis) of 11 cases (9 allergy and two autism patients) participating in the study were evaluated together.

Conclusions

Translating nutrigenetic and nutrigenomic research into multidisciplinary clinical practice is the most challenging aspect. It is now evident that integrating data regarding phenotype and genotype, and using nutrition, lifestyle, and supplements tailored to an individual's genetics can increase clinical success. Importantly, if we wish to adopt an epigenomic approach, we must incorporate analyses of nutrigenetics, microbiota, and personalized risk based on test results.

## Introduction

After the completion of the Human Genome Project in 2003, the concept of precision medicine and personalized medicine (PM) was introduced to the global medical community as a new field studying genetic variations. PM seeks to determine the genetic, phenotypic, and environmental factors of health risks. The 4Ps (Predictive, Preventive, Personalized, Participatory) of PM are defined as 'personalized prediction' in the diagnosis and treatment of chronic diseases, 'personalization' of medical preventive and treatment services for diseases, and active 'participation' of individuals, which brings the approach of 'personalized' medical practices instead of the same approach for everyone. Individual medicine/treatment is defined as foreseeing, preventing, personalizing treatment, and being participatory. The importance of PM in modern medicine and pharmacy is increasingly recognized [1, 2]. Precision/personalized medicine (PPM) and public health (PH) share a common goal. PPM presents scientists with the genetic building blocks of life. A population perspective is essential for PPM to succeed. Public Health Genomics promises a unified vision across scientific disciplines [3-6]. Following the Human Genome Project, the ENCyclopedia of DNA Elements (ENCODE), the Haplotype Map Project, and most recently, the EPIGENOME project were carried out to investigate the influence of environmental conditions on human phenotype and the significance of epigenetics in human health [7-9]. We stated that nutrigenetics is nutrition based on individual genes. These genetic differences, which are people's fingerprints, can reveal their genetic traits, and special nutrition programs can be prepared for individuals according to their genetic structures. Chief among these differences, as mentioned above, are single nucleotide polymorphisms (SNPs) gene expressions that occur as a result of a single nucleotide change [10]. Nutrigenomics identifies genome-wide interactions of gene expression using high-tech 'omics' technologies to investigate at the epigenome, genome, transcriptome, proteome, and metabolome levels how dietary nutrients alter the expression of gene clusters and thus affect human health [11, 12]. The science of nutrigenetics and nutrigenomics explores the relationship between nutrients and genes from basic science to clinical applications. By understanding how genes influence the body's response to nutrition or how nutrition alters the body's response to genetic coding variants, scientists have begun to unravel the code of good health. Personal profiling of genetic and nutritional responses can help determine which specific foods match an individual's DNA to produce the optimal biological response. The genomic structure of individuals directly impacts the control of their metabolism. Nutritional genomics provides insights into how individuals and populations can adapt their diets based on genetic differences. Personalized nutrition, mirroring its parallel in medical approaches, has introduced a new perspective to individual nutritional health with a 'personalized' approach using high-efficiency technologies [1,13]. For example, the importance of gene variants involved in 1-C metabolism in nutrigenomics on the epigenetic effect is significant: Methionine adenylyltransferase produces S-adenosylmethionine (SAM), a cofactor for many methylation reactions, such as DNA methylation, which affects gene regulation. Variations in folate (methylenetetrahydrofolate (MTHFR) substrate) and riboflavin (MTHFR cofactor) concentrations can modulate MTHFR gene activity [13].

Considering the critical role of the intestinal microbiome, the structures observed in the microbiome, alongside genetics, with the nutrigenomic perspective and the development of omic technologies in recent years, have revealed epigenetic changes caused by the microbiota, which are susceptible to degradation under the influence of environmental factors over time. It is possible to correct or prevent these variables with personalized medical practices [14]. It should be noted that personalized nutrition and medication use can significantly impact an individual's life. The use of genetic and microbiota tests is increasingly important for providing personalized prevention and safer drug choices in diseases such as cardiovascular diseases, diabetes, hypertension, autoimmune diseases, and cancer-conditions defined as chronic non-communicable diseases where epigenetic mechanisms play a crucial role [15]. It is believed that personalized nutrigenetic and microbiota test analyses for different complex diseases (cardiovascular, diabetes, hypertension, autoimmune diseases, depression, and cancer) may be more effective in identifying diseases due to negative epigenetic changes and in the selection of personalized nutrition and nutraceuticals [11,12,15]. Our aim in this study is to emphasize the importance of evaluating the nutrigenomic genetic risk factors, microbiota, and especially the VDR-microbiota relationship as a whole, as reflected in the phenotype.

## Materials and methods

In this study, genetic tests were performed on saliva samples from 24 patients aged between 7 and 57, and stool samples from 11 patients were used for microbiota testing. The patient group was selected from individuals who underwent genetic and microbiota testing at the Epigenetic Coaching Company. The patients' ages, genders, and the study itself were conducted in accordance with the principles of the Declaration of Helsinki. Approval was granted by the Ethics Committee of Biruni University's Molecular Biology and Genetics department, with the study approved under Ethics Committee Decision 2023/78-03.

Genotyping

Genomic DNA was isolated from buccal swab samples using a phenol/chloroform extraction method. The quality and concentration of the purified DNA were subsequently assessed using a NanoDrop™ 1000 spectrophotometer (Thermo Fisher Scientific Inc., Wilmington, DE, USA). Standardization of the DNA concentration to 50 microliters per milliliter was then performed. Analysis of the samples was subsequently conducted using a custom Infinium HTS iSelect microarray on an Illumina iScan instrument (Illumina, San Diego, CA, USA). Finally, single nucleotide polymorphism (SNP) alleles within the DNA were identified through the application of Illumina Genome Studio software version 2.0.5.

Microbiota analysis

In this research, fecal samples were collected and transported in a DNA/RNA Shield buffer medium to preserve the genetic material. Upon arrival at the laboratory, DNA was extracted directly from the stool samples using a commercially available Qiagen Power Soil DNA Extraction Kit (Qiagen, Hilden, Germany). Following extraction, the concentration of the isolated DNA was quantified using a NanoDrop device (Shimadzu, Japan). Next-generation sequencing of the full metagenome was performed using an Illumina NextSeq 500 platform (Illumina, San Diego, CA, USA) following the manufacturer's instructions. Libraries were processed to generate sequencing reads, with a minimum of 10 million paired-end reads produced per sample. Quality control procedures were implemented to analyze the sequencing data using the QIIME pipeline. Trimmomatic software was used to filter and trim the reads, ensuring a PHRED quality score of 30 or higher. Operational taxonomic units (OTUs) were defined using the Uclust method, and taxonomic classification was assigned using PyNAST against the GreenGenes database with an open-reference OTU picking approach. Scripting within the QIIME pipeline enabled the assessment of alpha and beta-diversity statistics. Finally, QIIME was used to generate graphical visualizations of the microbiota profiles using the map topological data analysis framework with the Bray-Curtis distance metric.

## Results

Analysis of nutrigenetic methylation test results

Among the heterozygous (CT) individuals who have the T risk allele of the MTHFR C677T (rs1801133) genetic variant, five have autism, three have allergies, and two have autoimmune thyroiditis. A person with allergies carries the homozygous (TT) genotype. In the MTHFR A1298C (rs1801131) genetic variant, 4 of the heterozygous (AC) individuals carrying the C risk allele have autism, three have allergies, and one has autoimmune thyroiditis. One person with autoimmune thyroiditis and 2 people with allergies carry the homozygous (CC) genotype (Table 1).

**Table 1:** Genetic polymorphisms in methylation. ASD: Autism Spectrum Disorder; AT: Autoimmune Thyroiditis; MTHFR: Methylenetetrahydrafolatreductase; TCN1: Transcobalamin 1; TCN2: Transcobalamin 2; MTRR: Methionine synthase reductase; PEMT: Phosphoethanolamine N-Methyltransferase.

Patient	Clinic	Sex	Age	Folate		B12					Choline
Genes				MTHFR C677T	MTHFR A1298C	TCN1	MTRR	MTHFR C677T	TCN2		PEMT
Rs -code				rs1801133	rs1801131	rs526934	rs1801394	rs1801133	rs9606756	rs1801198	rs7946
Minor Allele				T	C	G	G	T	G	G	T
OO	ASD	Male	23	CC	AC	AG	AG	CC	AA	CC	CT
TK	Allergy	Male	12	CT	AC	AG	AA	CT	AG	CG	CT
AU	ASD	Male	11	CT	AC	AA	AG	CT	AA	CG	TT
HDA	ASD	Male	29	CT	AA	AA	AA	CT	AG	CG	TT
ABO	ASD	Male	8	CT	AC	AA	AG	CT	AA	CG	CC
MAE	ASD	Male	37	CT	AA	AA	AG	CT	AA	CG	CT
ZJ	ASD	Male	10	CC	AA	AG	GG	CC	AA	GG	CT
AM	ASD	Male	7	CT	AA	AG	AG	CT	AG	CC	TT
SS	Allergy	Female	37	CT	AC	AG	AG	CT	AA	CG	CT
EY	AT	Female	45	CC	AC	AA	AG	CC	AA	CG	CT
CD	ASD	Female	12	CC	AC	AA	AG	CC	AA	CC	TT
YG	Allergy	Male	22	CC	CC	AA	AG	CC	AG	CC	CT
İA	AT	Female	57	CT	CC	AG	AG	CT	AA	CG	TT
YM	Allergy	Female	50	CC	AC	AG	AA	CC	AA	CG	TT
NS	Allergy	Female	54	TT	AA	AA	AG	TT	AG	CC	CT
HP	Allergy	Female	45	CT	AA	AG	GG	CT	AA	CG	TT
TP	Allergy	Female	12	CC	AA	AA	GG	CC	AG	CC	TT
NB	AT	Female	43	CT	AA	AA	GG	CT	AA	GG	CC
OE	Allergy	Male	32	CC	CC	AA	AG	CC	AA	CC	TT

Three autism patients are heterozygous in the Transcobalamin (TCN1) (rs526934-G) gene, six are heterozygous in the methionine synthase reductase (MTRR) (rs1801394-G) gene, and one is homozygous (GG) in the MTRR gene. Two autism patients are heterozygous (AG) in the TCN2 (rs9606756-G) gene, and four are heterozygous (CG) in the TCN2 (rs1801198-G). Four allergy patients carry the heterozygous (AG) genotype in the TCN1 genetic variant, four have the heterozygous (AG) genotype, and three allergy patients have the homozygous (GG) genotype in the MTRR polymorphism. Four allergy patients carry the heterozygous (AG) genotype in the TCN2 (rs1801198-G), and four have the heterozygous (CG) genotype in the TCN2 (rs9606756-G) polymorphisms. Only one autoimmune patient has a risk allele (AG) in the TCN1 variant, one has a heterozygous (AG) genotype in the MTRR variant, and one has a homozygous (GG) genotype. Two patients with autoimmune thyroiditis carry the heterozygous (CG) genotype, and one carries the homozygous (GG) genotype in the TCN2 polymorphisms. Table 1 presents the genotypes of 19 cases carrying genetic variants in Folate, B12, and Choline metabolism belonging to the study's patient group.

Analysis of nutrigenetics and microbiota test results

The microbiota of the study's patient group was evaluated using the Firmicutes/Bacteroides ratio, which is an indicator of diversity and dysbiosis. Table 2 presents the genotypes of 11 cases (9 with allergies and two with autism) carrying vitamin D receptor (VDR) genetic variants that affect the intestinal flora.

**Table 2:** VDR and microbiota. ASD: Autism Spectrum Disorder; VDR: Vitamin D receptor.

Patient	Clinic	VDR FokI Rs2228570 C	VDR BsmI Rs1544410 A	VDR TaqI Rs731236 C	VDR ApaI Rs797522 C	Microbiota Diversity (1-10)	Firmicutes/ Bacteroides rate
YG	Allergy	CC	AG	CT	AA	1.8 low	0.43 good
YM	Allergy	CC	AG	CT	AC	3.4 low	0.91 good
ZJ	ASD	AC	GG	TT	CC	7.5 medium	0.95 good
RZ	ASD	AC	GG	TT	AA	9.6 good	2.11 dysbiosis
DG	Allergy	AC	AG	CT	AC	2.2 low	3.62 dysbiosis
MG	Allergy	AC	GG	TT	CC	4.7 low	2.15 dysbiosis
AMT	Allergy	CC	AG	CT	AC	1.6 low	0.53 good
TA	Allergy	CC	GG	TT	AC	2.8 low	0.58 good
HP	Allergy	CC	AG	CT	AA	2.3 low	1.06 risk
TP	Allergy	AC	GG	TT	AA	6.7 medium	0.88 good
OE	Allergy	CC	AG	CC	AC	4.9 low	0.91 good

Considering the variant in the Phosphatidylethanolamine N-methyltransferase (PEMT) gene (rs7946-T), four of the autism patients have the homozygous (TT) genotype, and three have the heterozygous (CT) genotype. Among the allergy cases, four carry the heterozygous (CT) genotype, and four carry the homozygous (TT) genotype. It was observed that one patient with autoimmune thyroiditis had the heterozygous (CT) genotype and another had the homozygous (TT) genotype (Table 1).

It was observed that eight of these cases had low microbiome diversity, two had moderate, and one had good microbiome diversity. As a result of evaluating the Firmicutes/Bacteroides ratio, dysbiosis was observed in three of the cases; seven were good, and one was at risk. While one of the autism patients had medium and one had good microbiome diversity, it was observed that eight of the allergy patients had low and one had medium diversity (Table 2).

While 6 of the allergy patients carry the homozygous (CC) genotype in the VDR FokI (rs2228570-C) variant, three have the heterozygous (AC) genotype. Both autism patients are at risk of being heterozygous (AC) for the VDR FokI variant. While there is no risk allele in the VDR BsmI (rs1544410-A) variant for autism patients, six of the allergy patients carry the heterozygous (AG) genotype. Considering the VDR TaqI (rs731236-C) variant, five of the allergy patients have the heterozygous (CT) genotype, and only one case has the homozygous (CC) genotype. There is no risk of the VDR TaqI variant in autism patients. Five of the allergy patients had a heterozygous (AC) genotype in the VDR ApaI (rs797522-C) variant, and one carried a homozygous (CC) genotype. While one of the autism patients has no risk in the VDR ApaI variant, one has the homozygous (CC) genotype (Table 2).

Analysis of nutrigenetic VDR test results

Table 3 presents the genotypes of 19 cases carrying VDR genetic variants from the patient group participating in the study.

**Table 3:** VDR test results. ASD: Autism Spectrum Disorder; VDR: Vitamin D receptor.

Patient	Clinic	Gender	Age	VDR FokI	VDR TaqI	VDR BsmI	VDR ApaI
Rs-code				Rs2228570	Rs731236	Rs1544410	Rs797522
Minor Allele				C	C	A	A
OO	ASD	erkek	23	CC	CT	AG	AC
TK	Allergy	male	12	CC	CT	AA	AA
AU	ASD	male	11	CC	CT	AG	AC
HDA	ASD	male	29	CC	CC	AA	AA
ABO	ASD	male	8	CC	CT	AG	AC
MAE	ASD	male	37	AC	CT	AG	AC
ZJ	ASD	male	10	AC	TT	GG	CC
AM	ASD	male	7	AC	CT	AG	AA
SS	Allergy	female	37	CC	CT	AG	AC
EY	AT	female	45	AA	TT	GG	AC
CD	ASD	female	12	CC	CT	AG	AC
YG	Allergy	male	22	CC	CT	AG	AA
İA	AT	female	57	CC	TT	GG	CC
YM	Allergy	female	50	CC	CT	AG	AC
NS	Allergy	female	54	CC	TT	GG	AC
HP	Allergy	female	45	CC	CT	AG	AA
TP	Allergy	female	12	AC	TT	GG	AA
NB	AT	female	43	AC	TT	GG	CC
OE	Allergy	male	32	CC	CC	AG	AC

Eight cases were included in our study as autism patients, eight as allergy patients, and three as autoimmune thyroiditis patients. Five of the autism patients carry the homozygous (CC) genotype in the VDR FokI (rs2228570-C) variant, and three carry the heterozygous (AC) genotype. Seven of the allergy patients have the homozygous (CC) genotype, and one has the heterozygous (AC) genotype for the VDR FokI variant. One of the autoimmune thyroiditis patients is homozygous (CC), and one is heterozygous (AC) (Table 3).

Six of the autism patients have the heterozygous (CT) genotype in the VDR TaqI (rs731236-C) variant, and one has the homozygous (CC) genotype. Five of the allergy patients are at risk with the heterozygous (CT) VDR TaqI variant, and one carries the homozygous (CC) genotype. The risk allele was not found in this gene variant in patients with autoimmune thyroiditis. Six of the autism patients are heterozygous (AG), and one has a homozygous (AA) genotype in the VDR BsmI (rs1544410-A) polymorphism. In the VDR BsmI genetic variant, one of the allergy patients has the homozygous (AA) genotype, and five have the heterozygous (AG) genotype. There is no risk of this gene variant in patients with autoimmune thyroiditis (Table 3).

Considering the VDR ApaI (rs797522-C) genetic variant, five of the autism patients have the heterozygous (AC) genotype, and two have the homozygous (AA) genotype. Among the allergy patients, three have the heterozygous (AC) genotype, and four have the homozygous (AA) genotype. Among patients with autoimmune thyroiditis, the heterozygous (AC) genotype was observed in only one case (Table 3).

## Discussion

From a nutrigenetic and epigenetic perspective, the definition of phenotype and genotype is as follows: Genotype is the genetic code of individuals. Phenotype refers to a person's appearance, which is influenced by their genotype and environmental conditions. Although 99.99% of the genomic structure is the same across individuals, there are 0.01% individual genetic differences. These differences in phenotype lead to variations in bodies due to the interaction and change of foods, living conditions, lifestyle, and socio-cultural and economic levels. This is associated with differences in hair, skin color, height, and weight, as well as different microbiotas, intolerances, allergies, and diseases [11, 16].

In the field of Predictive, Preventive, Personalized, and Participatory Medicine (PM), when assessing individuals according to their lifestyle and genetic differences, a molecular approach based on nutrigenomic studies and a personalized diet can be recommended for early treatment [2]. When examining the connection between nutrigenomics and genetics, the relevant fundamentals include single carbon (1C) metabolism, transsulfuration, and methylation. These pathways are pivotal in regulating DNA expression. Essential in this mechanism, known as 1C metabolism, is folate metabolism, which serves to activate and transfer 1C units for biosynthetic processes that include the remethylation of homocysteine and the synthesis of purine and thymidine [17]. Disruptions in 1C metabolism due to genetic polymorphism of food-related enzymes play an essential role in the pathophysiology of epigenetic diseases, particularly cancer, through abnormal gene expression via DNA synthesis/repair and epigenetic mechanisms [18].

This investigation explored the potential association between genetic polymorphisms within genes encoding enzymes of the one-carbon metabolism pathway and susceptibility to various diseases. The one-carbon metabolism pathway plays a critical role in cellular methylation, a process involving the transfer of a methyl group for diverse biological functions. The focus of the study was on polymorphisms within five essential genes (MTHFR, MTRR, TCN1, TCN2, and PEMT) responsible for encoding enzymes in the methylation cycle. Notably, previous research has yet to comprehensively evaluate the contribution of all enzymes involved in this pathway. MTHFR, in particular, exhibits two well-characterized polymorphisms: C677T (rs1801133) and A1298C (rs1801131). The C677T variant is associated with elevated homocysteine levels, while the A1298C variant does not significantly impact enzyme activity. However, both variants may influence MTHFR's dependence on specific cofactors for optimal function. Existing literature suggests potential links between MTHFR polymorphisms and an increased risk of various pathologies such as cancer, cardiovascular diseases (CVDs), diabetes, inflammatory conditions, and vascular disorders [19]. Together with genetic and microbiota analyses, we examined factors that increase susceptibility to diseases reflected in the phenotype. Although the number of cases is limited, we believe that our study will shed light on future research on personalized case definitions from phenotype to genotype, including conditions like neural tube defects, pregnancy complications, neurodegenerative disorders, mood disturbances, peripheral vascular disease, malignancies, allergies, neurodevelopmental disorders, and autoimmune conditions. MTHFR catalyzes the conversion of 5,10-methylenetetrahydrofolate (5,10-methylene THF) to L-methylfolate, a crucial methyl donor in the methylation cycle. This methyl group is subsequently utilized by MTR and MTRR to generate S-adenosylmethionine (SAM), the universal methyl donor for numerous methylation reactions in the human body [19, 20].

When we look at the SNP related to 1C metabolism in the patients we encountered clinically, disorders in methionine metabolism or folate have been identified in many individuals with ASD, which is a significant public health problem. Some studies indicate that the folate-methionine cycle may play an important role in the pathogenesis of autism. Several studies have shown that low levels of vitamin B12, high levels of homocysteine, and low levels of folate are associated with ASD. These changes in serum metabolites may result from malnutrition as well as genetic tendencies such as polymorphisms in the MTHFR gene [21]. Based on the relationship between common genetic variants and ASD, comprehensive studies have shown that MTHFR polymorphisms are associated with an increased risk of ASD [22-24]. In a meta-analysis examining the relationship between ASD and the MTHFR C677T/A1298C, it was stated that C677T is a risk factor, but A1298C is not a risk factor for ASD [23]. Another meta-analysis study looking at common genetic variants with ASD stated that both variants of MTHFR, namely C677T and A1298C, increased the risk of autism [24]. In the study conducted by Mohammed et al., it was shown that the presence of the MTHFR 677T allele (rs1801133) and the T allele frequency was higher in autistic children compared to healthy controls, and increased the risk of autism by 2.79 times. The 1298C (rs1801131) allele frequency alone was not associated with risk. However, an 8.11-fold risk effect was reported with the cumulative effect of two polymorphisms of MTHFR (677CT+TT/1298AC+CC coexistence), meaning that A1298C has only an additive effect [22]. In our study, while 5 of the autism cases were found to be heterozygous (CT) for the T risk allele of the MTHFR C677T genetic variant, four autism patients were found to carry the heterozygous (AC) genotype of the MTHFR A1298C genetic variant. Two autism patients were carrying risk alleles, both variants heterozygous (MTHFRC677T-CT and MTHFRA1298C-AC) (Table 1). As a result of meta-analyses, genetic polymorphisms of the folate pathway were found to be moderate predictors of autism risk. Synergistic interactions between MTHFR C677T (rs1801133) and MTRR A66G (rs1801394) increase homocysteine [25]. To evaluate the effect of the MTRR 66 A→G (rs1801394) polymorphism on total plasma homocysteine (tHcy), a study found that the MTRR genotype has a significant impact on the ranking and that the rs1801394-AA genotype contributed to a moderate increase in tHcy levels throughout the distribution. It provides the first evidence that it significantly affects tHcy concentration [26]. A case-control study found that the rs1801394 A allele in the MTRR gene was associated with a reduced risk of ASD [22]. In our study, genetic variants with a risk of increased homocysteine and genetic variants with a risk of ASD were detected in cases with ASD. Genetic variant distribution in ASD cases: While six autism cases had a heterozygous (AG) genotype in the MTRR gene (rs1801394), one case had a homozygous (GG) genotype in the MTRR gene (Table 1).

ASD patients have been found to have lower serum vitamin B12 compared to healthy controls [21, 27]. Polymorphisms seen in TCN1 (rs526934), TCN2 (rs9606756-rs1801198) and MTRR (rs1801394) genes cause low B12 levels [28-30]. The symptomatic improvement effect of B12 use in children with ASD has been demonstrated [31]. In our study, three of the autism patients were heterozygous (AG) in the TCN1 (rs526934) gene, six were heterozygous (AG) in the MTRR (rs1801394) gene, and one was a homozygous (GG) genotype in the MTRR gene. Four autism patients have the heterozygous (CG) genotype, and one has the homozygous (GG) genotype in the TCN2 (rs1801198-G) gene (Table 1). In a study, it was determined that the genetic variant homozygous TCN2 776GG (rs1801198) was more common in autistic children than in controls, and the GG variant was associated with a 1.7-fold increase in the risk of autism [32]. While four of the autism cases in our study carried the G risk allele heterozygous, one case had the homozygous (GG) variant. In our study, two autism patients have the heterozygous (AG) genotype in the TCN2 (rs9606756-G) gene (Table 1).

Choline is the precursor to betaine and betaine-derived methyl groups used for SAM-dependent methylation reactions, including membrane phosphatidylcholine (PC) synthesis. Choline can be obtained both externally from foods and synthesized de novo in the liver. Phosphatidylethanolamine methyltransferase (PEMT) enzyme activity is vital in liver synthesis. Since variants in the PEMT gene reduce enzyme activity, the choline needs of these individuals increase even more. In a study, it was stated that the rs7946 polymorphism caused a loss of function in the PEMT enzyme and that the diet was inadequate in terms of choline with this variant [33]. Nutrition monitoring to determine whether dietary intake of choline and betaine in children with autism is adequate to meet nutritional needs based on national recommendations has shown that 60-93% of children with ASD consume less than the recommended Adequate Intake (AI) for choline, resulting in low plasma levels. It was concluded that it was reflected [34]. When we look at the variant in the PEMT gene (rs7946-T) in our study, four of the autism patients have the homozygous (TT), and three of them have the heterozygous (CT) genotype (Table 1).

MTHFR genotypes are important in studies on the relationship between 1C metabolism and allergic diseases. Studies have shown that the TT genotype of the MTHFR C677T (rs1801133) polymorphism is associated with an increased risk of asthma. The MTHFR C677T genotype is also associated with folic acid concentration [35, 36]. Plasma folate levels are more affected in the TT genotype; that is, they show an increase in supplement use and a decrease in Hcy levels. A study stated that the CT or TT genotypes of the MTHFR C677T (rs1801133) polymorphism may be associated with a reduced risk of atopic asthma in school-age children, especially when exposed to high levels of folate and vitamins B2 and B6. Folate plays a role in DNA methylation through SAM formation in the Methylation pathway and thus affects the pathogenesis of asthma [37]. In another study, the relationship between the MTHFR C677T (rs1801133) genotype, plasma total homocysteine, and dietary intake of methionine, folate, vitamins B12, B6, and B2, as markers of atopy and impaired folate metabolism were examined. Atopy prevalence was found to be associated with the MTHFR C677T genotype. Since TT individuals have a higher risk of atopy than CC/CT individuals. It is thought that impaired folate metabolism may be associated with the development of atopy [38]. In our study, three allergy cases with the T risk allele of the MTHFR C677T (rs1801133) genetic variant carried the heterozygous (CT) genotype. In contrast, a homozygous (TT) genotype was observed in one case with an allergy clinic (Table 1).

The most common disease in endocrine system disorders is thyroid disorders [39]. MTHFR polymorphisms are associated with thyroid dysfunction. In a study investigating the relationship between subclinical hypothyroidism (SCH) and MTHFR gene polymorphisms, the MTHFR A1298C (rs1801131) polymorphism was not found to be a risk factor. However, the T allele frequency in the MTHFR C677T (rs1801133) polymorphism was found to be significantly higher in the SCH group patients compared to controls [40]. In our study, 2 of the patients with autoimmune thyroiditis were found to have a heterozygous (CT) genotype for the MTHFR C677T (rs1801133-T) polymorphism. In the MTHFR A1298C (rs1801131-C) variant, one case carries a heterozygous (AC), and the other case carries a homozygous (CC) genotype (Table 1). Another study examined the correlation between thyroid diseases and polymorphisms in the MTHFR C677T (rs1801133) and MTRR A66G (rs1801394) genes, and found that the T allele in MTHFR and the G allele in MTRR increased the risk of thyroid disorders [39]. Two of the autoimmune patients have a heterozygous (AG) genotype, and one has a homozygous (GG) genotype in the MTRR (rs1801394) variant (Table 1).

Although vitamin D's role in bone health is known, increasing evidence shows that vitamin D is a secosteroid that plays a role in cell differentiation, proliferation, vascular muscle functions, and vascular and metabolic health. Since the detection of vitamin D receptors, particularly on T cells, local production of active vitamin D has also been demonstrated in immune cells, increasing interest in the clinical implications of vitamin D status in immunity against autoimmune/inflammatory and infectious diseases. Vitamin D interacts with active vitamin D (calcitriol or 1,25(OH)2D3) and the VDR/retinoic acid promoter, leading to the transcription of more than 3000 genes in humans, including some genes involved in immune system functions [41,42].

Considering the research on VDR, which plays a vital role in the epigenetic mechanisms of vitamin D, the number of studies related to VDR polymorphism is increasing daily. One such disease is autism. When examining autism and VDR polymorphism, it was found that the C allele of the VDR (VDR taqI) rs731236 polymorphism was significantly associated with an increased risk of autism, while the G allele of the VDR (ApaI) rs7975232 polymorphism could be a protective factor against the development of autism [43]. Six of the autism patients have the heterozygous (CT) genotype in the VDR TaqI (rs731236) variant, and one has the homozygous (CC) genotype. Considering the VDR ApaI (rs7975232) genetic variant, 5 of the autism patients have the heterozygous (AC) genotype, and 2 have the homozygous (AA) genotype (Table 3).

Since conflicting results were encountered when examining VDR polymorphism and its relationship with autoimmune thyroid diseases, a comprehensive meta-analysis of eleven case-control studies was conducted to better understand the role of four polymorphisms in the development of Hashimoto's thyroiditis (HT). The results of this meta-analysis showed that only the FokI rs2228570 polymorphism was significantly associated with the risk of HT [44]. Another study conducted with patients with autoimmune thyroid disease and healthy controls found a relationship between the VDR FokI rs2228570 CC and CT genotypes and susceptibility to autoimmune thyroid diseases [45]. In our study, one of the autoimmune thyroiditis patients was homozygous (CC), and one was heterozygous (AC) genotype in the FokI (rs2228570) gene (Table 3).

According to the results of a meta-analysis of the relationships between allergic diseases and VDR gene polymorphisms, positive results were confirmed for the rs1544410 BsmI polymorphism in both atopic dermatitis and asthma, and the rs731236 TaqI polymorphism in atopic dermatitis. These studies have shown significant associations with allergic diseases in subgroups of participants by ethnicity for the rs7975232 ApaI polymorphism in Caucasians, the rs1544410 BsmI polymorphism in Caucasians and Asians, and the rs731236 TaqI polymorphism in Asians. When examining the relationships between allergic diseases and the VDR rs2228570 FokI polymorphism, no positive result was detected for this polymorphism [46]. In a study conducted in the Turkish population on VDR polymorphism and atopy, it was determined that there was no significant relationship between the FoqI Rs2228570 C risk allele, TaqI Rs731236 C risk and Apal Rs797522 T risk allele with atopic dermatitis susceptibility. However, it was determined that the BsmI Rs1544410 A risk allele polymorphism increased the risk [47]. In our study, seven of the allergy patients have the homozygous (CC) genotype and one has the heterozygous (AC) genotype in the VDR FokI (rs2228570) variant. While five of the allergy patients have the heterozygous (CT) genotype in the VDR TaqI (rs731236) variant, one carries the homozygous (CC) genotype. In the VDR BsmI (rs1544410) genetic variant, one of the allergy patients has the homozygous (AA) genotype, and five have the heterozygous (AG) genotype. When looking at the VDR ApaI (rs7975232) genetic variant, five of the allergy patients have the heterozygous (AC) genotype, and four have the homozygous (AA) genotype (Table 3).

VDR is a vital host factor that can influence the gut microbiome at the genetic level. The effect of vitamin D on the gut microbiota through VDR activation is closely related. Some studies have shown that vitamin D causes changes in the composition of the microbiome and that vitamin D deficiency leads to dysbiosis. Through reduction and stimulation of the microbiota, the Firmicutes/Bacteroides ratio has shown changes [48,49]. In our study, as a result of the evaluation of the Firmicutes/Bacteroides ratio, dysbiosis was observed in three of the cases, while seven were good, and one was at risk. One of the autism patients had medium microbiome diversity and one had good microbiome diversity. It was observed that eight of the allergy patients had low microbiome diversity and one had medium diversity (Table 2). When examining the effects of imbalances in the intestinal microbiota on diseases, the impact of environmental and microbial interactions on adaptive immune responses and allergic disease comes to the fore. A wide range of factors, including nutritional inputs, environmental, and genetic factors, can modulate the gut immune-microbiome axis and affect the occurrence of allergy, with dysbiosis of the microbiome leading to impaired immune modulation and the development of allergic diseases and further expansion of T helper 2 (Th2) inflammatory cells [50]. In our study, eight out of 11 cases (9 allergy and two autism patients) had low microbiome diversity, two had medium, and one had good microbiome diversity (Table 2).

There is increasing interest in the gut microbiota as a possible risk factor in the development of ASDs. It emphasizes the reciprocal communication between the gut and the brain (i.e., the so-called “gut-brain axis”). Evidence has shown that changes in the gut microbiota in children with autism are a link between both neurobehavioral and gastrointestinal symptoms. Studies have shown that gut dysbiosis has been widely evidenced in ASD; however, there is no single, distinctive profile regarding the composition of the microbiota in individuals with ASD. Administration of probiotics (primarily a mixture of Bifidobacteria, Lactobacilli, Streptococci, and Bifidobacteria) is the most promising treatment for neurobehavioral symptoms and intestinal dysfunction, but clinical studies are still limited and heterogeneous [51]. Data collected from recent studies indicate that probiotic treatment can increase vitamin D, VDR expression, and VDR activity in the host. It was reported that oral supplementation with L. reuteri NCIMB 30242 increased circulating 25(OH)D levels [52,53]. In another study, probiotic Lactobacillus GG and *Lactobacillus plantarum* (LP) increased VDR protein expression and have been shown to increase VDR transcriptional activity [54].

In our study, we examined together genetic and microbiota analyses that increase susceptibility to diseases reflected in the phenotype. Although the number of cases is limited, we believe that our study will shed light on examining genetic and microbiota results together in personalized approaches from phenotype to genotype.

## Conclusions

Translating nutrigenetic and nutrigenomic research into multidisciplinary clinical practice is the most challenging. It is now evident that integrating data regarding genotype and phenotype, and using nutrition, lifestyle, and supplements appropriate to the genetics of individuals will increase clinical success. What is important here is that if we want to adopt an epigenomic approach, we should make personalized supplement and nutrition recommendations based on nutrigenetics, microbiota, and personalized risk analyses derived from test results. In our study, we wanted to emphasize how variants of genes encoding enzymes involved in 1C metabolism, VDR genetic variants, and microbiota analysis results affect the phenotype.

PPM generates significant investments in population sciences. PH has a “collective” perspective focused on health promotion, prevention, and personal empowerment in disease/disability management. PPM adopts a “big data” perspective, emphasizing accountability, integrative approaches, and datafication. We believe that, based on precision medicine, genetic predispositions and microbiota analyses that cause diseases will provide a tool to prevent diseases and reduce the symptoms of diseases.
