# Correction: The Importance of Nutrigenetics and Microbiota in Personalized Medicine: From Phenotype to Genotype

**DOI:** 10.7759/cureus.c185

**Published:** 2024-07-10

**Authors:** Gulsen Meral, Elif S Aslan, Verda Tunaligil, Neval Burkay, Esma Gökcen Alper Acar, Muhammed Yunus Alp

**Affiliations:** 1 Molecular Biology and Genetics, Biruni University, Istanbul, TUR; 2 Molecular Biology and Genetics, Epigenetic Coaching, Norwich, GBR; 3 Epidemiology and Public Health, Ministry of Health, Istanbul Health Directorate, The Medical Device and Simulation Center (SIMMERK5), Istanbul, TUR; 4 Nutrition, Epigenetic Coaching, Norwich, GBR; 5 Medical Biology, Merzifon Karamustafa Paşa Devlet Hastanesi, Amasya, TUR; 6 Genetics, Epigenetic Coaching, Norwich, GBR

This article has been corrected as follows to replace reference 47 with the correct information.

 

Original reference: 

Mostowska A, Lianeri M, Wudarski M, Olesińska M, Jagodziński PP: Vitamin D receptor gene BsmI, FokI, ApaI and TaqI polymorphisms and the risk of systemic lupus erythematosus. Mol Biol Rep. 2013, 40:803-810. 10.1007/s11033-012-2118-6

New reference: Kılıç S, Sılan F, Hız MM, Işık S, Ögretmen Z, Özdemir Ö. Vitamin D receptor gene BSMI, FOKI, APAI, and TAQI polymorphisms and the risk of atopic dermatitis. J Investig Allergol Clin Immunol. 2016, 26:106-110. 10.18176/jiaci.0020

The authors and journal deeply regret that this error was not identified and addressed prior to publication.

 

